# The effect of enhanced acetate influx on *Synechocystis* sp. PCC 6803 metabolism

**DOI:** 10.1186/s12934-017-0640-x

**Published:** 2017-02-02

**Authors:** Kati Thiel, Eerika Vuorio, Eva-Mari Aro, Pauli Tapio Kallio

**Affiliations:** Molecular Plant Biology, Department of Biochemistry, University of Turku, Itäinen Pitkäkatu 4 C, 6th Floor, 20014 Turku, Finland

**Keywords:** *Synechocystis* sp. PCC 6803, Heterologous acetate transporter, ActP, Facilitated acetate influx, Enhanced growth, Glycogen metabolism, Inhibition of photosynthesis

## Abstract

**Background:**

Acetate is a common microbial fermentative end-product, which can potentially be used as a supplementary carbon source to enhance the output of biotechnological production systems. This study focuses on the acetate metabolism of the photosynthetic cyanobacterium *Synechocystis* sp. PCC 6803 which is unable to grow on acetate as a sole carbon source but still can assimilate it via acetyl-CoA—derived metabolic intermediates. In order to gain insight into the acetate uptake, associated limitations and metabolic effects, a heterologous acetate transporter ActP from *Escherichia coli* was introduced into *Synechocystis* to facilitate the transport of supplemented acetate from the medium into the cell.

**Results:**

The results show that enhanced acetate intake can efficiently promote the growth of the cyanobacterial host. The effect is apparent specifically under low-light conditions when the photosynthetic activity is low, and expected to result from increased availability of acetyl-CoA precursors, accompanied by changes induced in cellular glycogen metabolism which may include allocation of resources towards enhanced growth instead of glycogen accumulation. Despite the stimulated growth of the mutant, acetate is shown to suppress the activity of the photosynthetic apparatus, further emphasizing the contribution of glycolytic metabolism in the acetate-induced effect.

**Conclusions:**

The use of acetate by the cyanobacterium *Synechocystis* sp. PCC 6803 is at least partially restricted by the import into the cell. This can be improved by the introduction of a heterologous acetate transporter into the system, thereby providing a potential advantage by expanding the scope of acetate utilization for various biosynthetic processes.

**Electronic supplementary material:**

The online version of this article (doi:10.1186/s12934-017-0640-x) contains supplementary material, which is available to authorized users.

## Background

The ability of microbial hosts to utilize acetate as an auxiliary carbon source is of potential biotechnological interest: in some biological contexts acetate is a waste product, while under other conditions it can be used as a building block to facilitate growth and biosynthesis of desired end-products. Essentially, acetate becomes dispensable waste whenever it cannot be further oxidized in aerobic respiration, or if the uptake via acetyl-CoA and direct use as a biosynthetic precursor is restricted. Understanding the associated processes and the species-specific limitations is thus of scientific and biotechnological value to enable efficient end-product recycling and process optimization. Acetate which could be used for such applications is generated in large quantities through anaerobic digestion in the primary treatment of wastewater sludge [1, 2], as a bi-product in a range of industrial processes, or it could potentially be produced via microbial conversion of CO derived from industrial waste gas [3]. From this perspective, the current work is focused on engineering the acetate metabolism of a well-established cyanobacterial model species *Synechocystis* sp. PCC 6803 (*Synechocystis* from here on), which has been extensively studied as a prominent autotrophic host for the photosynthetic production of desired compounds directly from CO_2_ and water. Although the key genes and pathways involved in acetate uptake can be identified based on the existing metabolic models, the available literature on acetate metabolism in *Synechocystis* is rather limited. It is unclear to what extent acetate could serve as a supplement to enhance the efficiency of the cyanobacterial production systems, and thus to contribute to the development of commercially competitive strategies in the future.

Acetate is produced by different species of photosynthetic cyanobacteria, including *Synechocystis*, which are adapted to sustain also heterotrophic growth and harbor pathways for fermentative metabolism to cope with anaerobic or temporarily anoxic environments [4, 5]. The utilization of acetate as a carbon source, on the other hand, is limited to those organisms which are capable of incorporating acetate into the central carbon metabolism through the glyoxylate pathway (glyoxylate shunt) and gluconeogenesis. The glyoxylate shunt is a detour of the oxidative tricarboxylic cycle (TCA) which bypasses the two decarboxylative reactions, thus allowing the use of two-carbon units for the assembly of four-carbon precursors needed for gluconeogenesis. The unicellular green alga *Chlamydomonas*, for example, harbors this capacity, and supplemented acetate can be used directly as a substrate for heterotrophic growth and production of desired target products. *Synechocystis*, however, cannot grow on acetate alone as it lacks the two glyoxylate shunt enzymes, isocitrate lyase (EC 4.1.3.1) and malate synthase (EC 2.3.3.9) required for acetate assimilation [6–8]. This obviously limits the use of acetate for biotechnological applications, but also calls for more comprehensive understanding of the associated metabolism in *Synechocystis*.

Bacteria which are unable to produce carbohydrates via gluconeogenesis, can still assimilate acetate by conversion into acetyl-CoA, a common intermediate and a precursor for various primary and secondary metabolic pathways. In *Synechocystis* the activation of acetate into acetyl-CoA is primarily catalyzed by ATP-driven acetyl-CoA synthetase (ACS encoded by sll0542) or alternatively, the biosynthesis may proceed through the combined action of acetate kinase (ACK encoded by sll1299) and phosphotransacetylase (PTA encoded by slr2132) [9]. Acetyl-CoA can be used as a building block for protein synthesis through incorporation into α ketoglutarate derived amino acids (glutamate, glutamine, proline, arginine) [7] or depending on the conditions, directly as a substrate for lipid biosynthesis [10]. In addition, acetyl-CoA serves as a precursor for the biosynthesis of secondary metabolites such as terpenoids, flavonoids or polyketides [10]. In *Synechocystis* acetate has been reported to increase the intracellular pool of pyruvate available for biosynthetic reactions [10], and lead to enhanced production of commercially interesting end-products such as polyhydroxybutyrate (PHB) [11–13] and d-lactic acid [7]. In regards to growth, supplemented acetate has been reported to have either no effect [7] or a slight stimulating effect [11] in *Synechocystis*.

Some organisms which have evolved to efficiently utilize acetate for growth have specific transport systems to facilitate the intake of acetate from the surrounding environment into the cell. For example, *Escherichia coli* expresses a homodimeric acetate/glycolate transporter ActP on the inner membrane [14, 15], providing an advantage when extracellular acetate concentrations are low and intake may limit the carbon flux to central metabolism. In cyanobacteria, however, specific acetate transporters have not been identified, and acetate is generally expected to enter the cells without any active protein-mediated transport [10, 16]. The intake has been reported to be dependent on pH, light and CO_2_ levels, and at µM concentrations the acetate transport into the cell per se is not proposed to be the limiting factor [10].

Currently there is inconclusive knowledge regarding the cellular intake of acetate and the limitations in the associated mechanisms through which acetate can be incorporated into the central metabolism in *Synechocystis*. The goal of this study was to provide a more comprehensive understanding of the potential of acetate utilization, and to evaluate if an augmented acetate intake could be beneficial for autotrophic production platforms. The approach was to introduce a heterologous acetate transporter enzyme ActP from *E. coli* into *Synechocystis*, followed by evaluation of phenotypic growth effects and oxygen evolution capacity, in combination with biochemical quantitation of selected key metabolites (including acetate, glycogen and PHB) and reactive oxygen species (ROS).

## Results

### Heterologous ActP acetate transporter over-expression construct for *Synechocystis*

Sequence homology comparison (CyanoBase) did not reveal feasible candidates coding for putative native acetate transporter proteins in *Synechocystis*, supporting the view that the intake takes place via spontaneous diffusion through the cell membrane. With the aim of enhancing the acetate influx into the cyanobacterial host, a characterized acetate transporter ActP from *E. coli* [14] was selected as a heterologous over-expression target. The gene *yjcG* encoding for ActP was subcloned from *E. coli* K-12 (substrain MG1655) into an autonomously replicating expression shuttle vector pDF-lac under the control of a tunable lac-promoter [17] and confirmed by sequencing. The construct was then transformed into *Synechocystis*, and the presence of the target gene in resulting spectinomycin/streptomycin-resistant clones was verified with colony PCR (Additional file 1).

### ActP enhances growth of *Synechocystis* in the presence of acetate

**Fig. 1:**
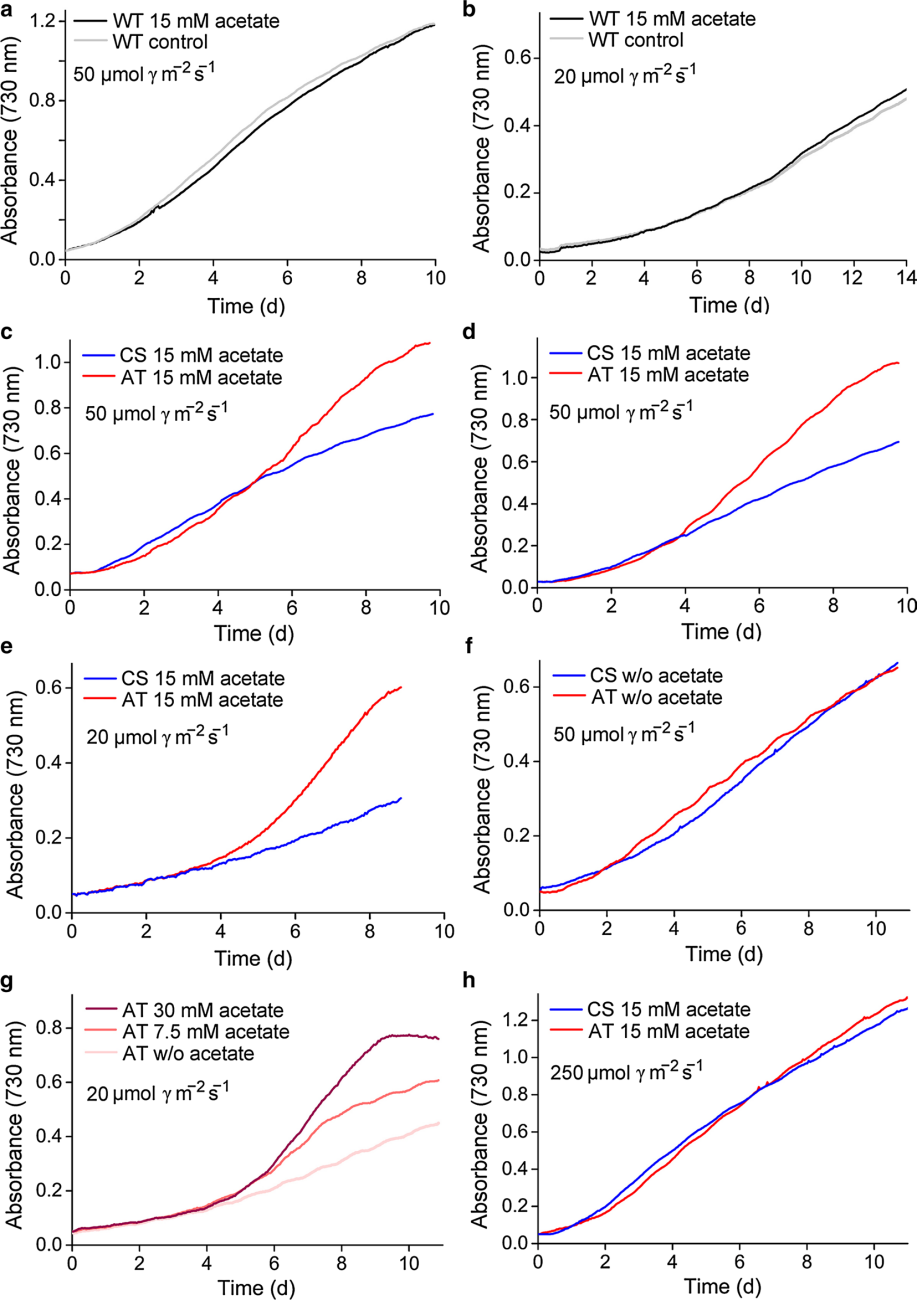
Growth curves of different *Synechocystis* sp. PCC 6803 strains cultivated in photobioreactor under varying conditions. Wild type strain (WT) grown in the presence of 15 mM supplemented acetate (*black line*) and in the absence of acetate (*grey line*) **a** under continuous light of 50 μmol photons m^−2^ s^−1^ for 10 d and **b** under continuous light of 20 μmol photons m^−2^ s^−1^ for 14 days. Acetate transporter expression strain (AT; *red line*) and control strain (CS; *blue line*) grown with 15 mM supplemented acetate starting from **c** OD_750_ = 0.1 and **d** OD_750_ = 0.05 under continuous light of 50 μmol photons m^−2^ s^−1^ for 10 days. **e** AT and CS grown under continuous light of 20 μmol photons m^−2^ s^−1^ for 9 days with 15 mM supplemented acetate. **f** AT and CS grown in the absence of acetate or IPTG induction under continuous light of 50 μmol photons m^−2^ s^−1^ for 11 days. **g** AT cultivated with supplemented with 30 mM acetate (*dark red line*), 7,5 mM acetate (*red line*) and no acetate (*light red line*) under continuous light of 20 μmol photons m^−2^ s^−1^for 11 d. **h** AT and CS grown for 11 days under continuous light of 250 μmol photons m^−2^ s^−1^ with 15 mM supplemented acetate. All cultures were started from OD_750_ = 0.05, and AT and CS were grown in the presence of IPTG, unless stated otherwise. AT and CS were always cultivated with 25 µg/mL Spec and 12,5 µg/mL Str to maintain the selection pressure for the expression plasmid (**c–h**), which resulted in slower overall growth in comparison to WT cultured without antibiotics (**a**, **b**). In each case, the graphs are representatives of at least three parallel biological replicate cultures, with the exception of the negative controls in **g** and **f** with two replicates (see Additional file 2 for more comprehensive set of the growth curves)

**Fig. 2:**
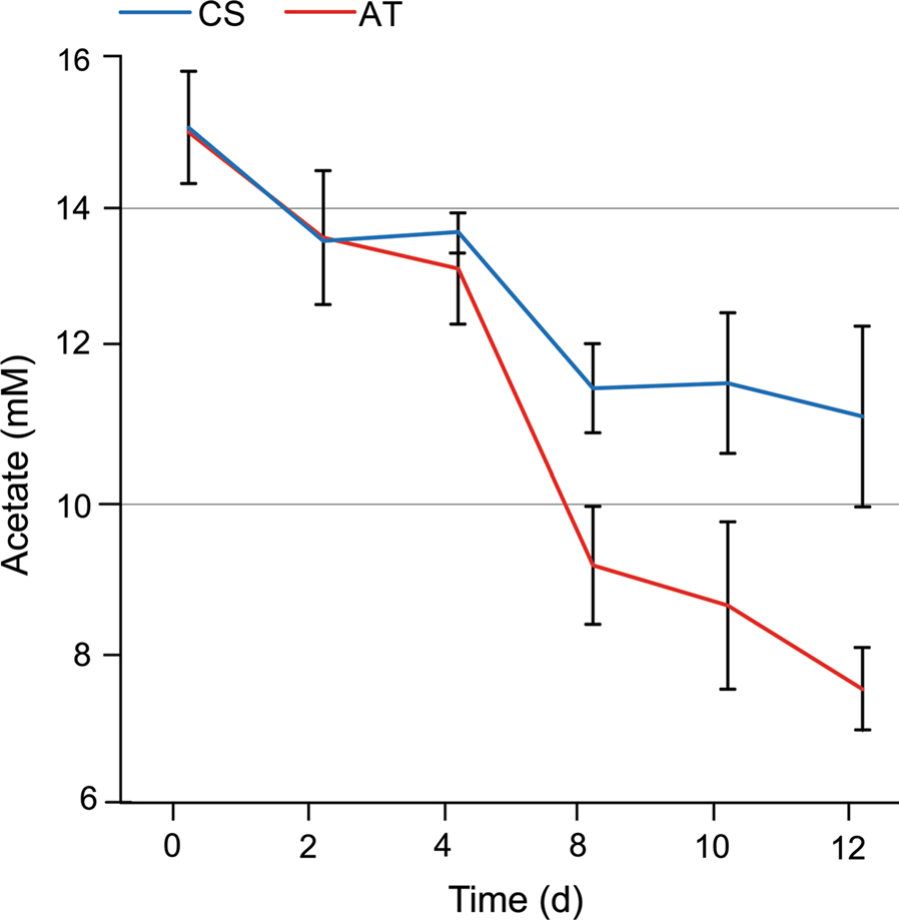
Acetate consumption by the cyanobacterial strains. Quantitative determination of acetate consumption by the *Synechocystis* sp. PCC 6803 acetate transporter expression strain (AT; *red line*) and the control strain (CS; *blue line*) monitored over a 10 days batch cultivation period in the presence of supplemented 15 mM acetate under continuous light of 20 μmol photons m^−2^ s^−1^ (starting OD_750_ = 0.05). Acetate concentration was measured as mM in the growth media. The mean and standard deviation represent three biological replicates

Growth of the *Synechocystis* acetate transporter expression strain (AT) was evaluated in comparison to the control strain (CS) harboring a corresponding empty plasmid under different intensities of constant light, in the presence and absence of acetate (Fig. 1; Additional file 2). The cultivations were conducted using a MC1000 photobioreactor equipped with eight parallel batch culture chambers with independent light sources and capacity for automated continuous monitoring of optical density (OD) at 730 nm. The cultures were typically carried out in at least three biological replicates (see Additional file 2) and two independent repetitions, and although the growth was relatively sensitive to variations in light intensity and medium composition, the observed trends were repeatable between parallel trials. Supplementation of 15 mM acetate into the BG-11 medium did not have any effect on the growth of the wild type *Synechocystis* under continuous low light of 50 μmol photons m^−2^ s^−1^ (Fig. 1a) or 20 μmol photons m^−2^ s^−1^ (Fig. 1b) and atmospheric CO_2_. In contrast, the growth of the AT mutant was enhanced under corresponding conditions (Fig. 1c–e). No difference was observed between CS and the AT mutant in the absence of acetate or induction by IPTG (Fig. 1f; Additional file 2, pane i), demonstrating that the phenotypic growth enhancement was directly linked with the over-expressed acetate transporter. The maximum growth rate of the AT strain correlated with the amount of supplemented acetate (Fig. 1g), and within the 0–30 mM concentration range used in the experiments, acetate did not have any direct impact on the culture medium pH. The phenotypic growth-enhancement of the AT mutant was never observed at the beginning of cultivation but it typically became apparent around the day four (Fig. 1c–e, g). This delay appeared to be independent of the initial culture cell density (Fig. 1c, d), light intensity (Fig. 1d, e) or the amount of acetate supplied (Fig. 1g). As seen in Fig. 2, consumption of acetate from the medium coincided with the enhanced growth of the mutant, with the difference between AT and CS becoming clear only after day four (Fig. 2).

### ActP enhances growth specifically under low-light conditions

The phenotypic growth enhancement observed for the AT mutant in the presence of acetate was dependent on the light intensity, and significantly declined upon the shift towards high-light. The clearest impact was observed under low continuous light (20 μmol photons m^−2^ s^−1^), where the maximum difference in the growth between the AT mutant and CS was almost tenfold (as estimated based on the growth-curve slopes) (Fig. 1e). In comparison, under moderately higher light (50 μmol photons m^−2^ s^−1^) the benefit was less profound (Fig. 1c, d), while under high light (250 μmol photons m^−2^ s^−1^) the phenotype disappeared completely and the two strains could not be distinguished based on the growth profiles (Fig. 1h).

### Increased growth is associated with changes in glycogen metabolism

**Fig. 3:**
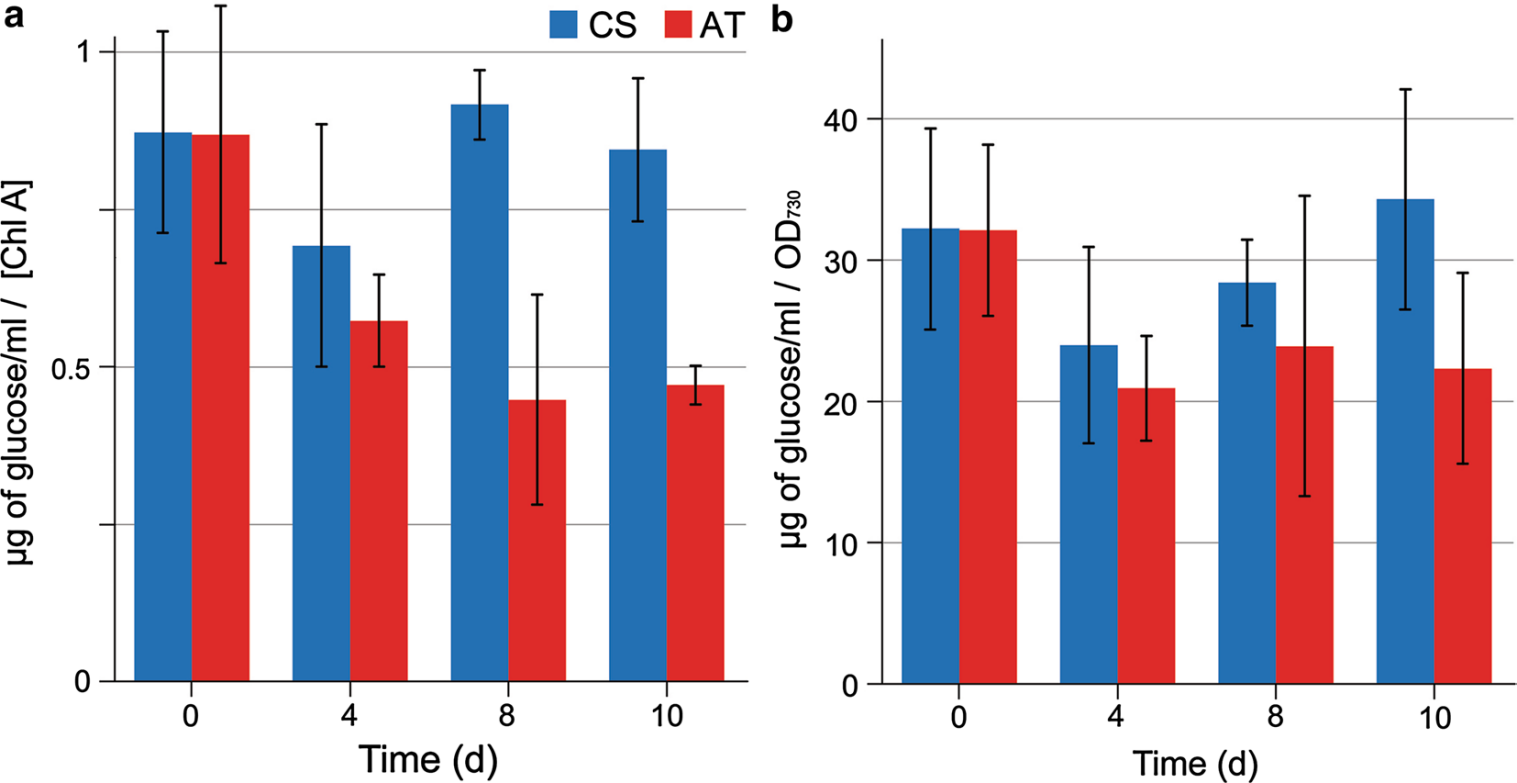
Glycogen content of the cyanobacterial strains. Quantitative determination of glycogen in the *Synechocystis* sp. PCC 6803 acetate transporter expression strain (AT; *red bars*) and the control strain (CS; *blue bars*) cultivated in photobioreactor MC1000 in the presence of supplemented 15 mM acetate for 10 d under continuous light 20 μmol photons m^−2^ s^−1^ (starting OD_750_ = 0.05) (see Fig. 1e). The glycogen was broken to glucose and quantified in respect to **a** chlorophyll *a* content and **b** cell density OD_730_. The mean and standard deviation represent three biological replicates

In order to study the possible connection between the observed growth enhancement and glycogen metabolism, the intracellular glycogen content of the AT mutant and the CS was quantitated and compared at different time points (Fig. 3a, b). After the initial drop observed for both strains, the overall glycogen levels of the CS resumed over the 10-day cultivation period under 20 μmol photons m^−2^ s^−1^ continuous light in the presence of acetate, unlike in the AT mutant (Fig. 3). During the phase of enhanced growth (see Fig. 1e; day 4 onwards), the glycogen content of the AT mutant either decreased (Fig. 3a) or remained unchanged (Fig. 3b), depending on the normalization method used. The CS, in contrast, continued to accumulate glycogen over this period.

### Enhanced acetate intake does not alter PHB metabolism

To evaluate whether the phenotypic growth effect of the AT mutant was accompanied by changes in PHB metabolism, the intracellular PHB levels of the mutant strain and CS were compared in an eight-day MC1000 cultivation (20 μmol photons m^−2^ s^−1^). When the cells were cultured in the absence of acetate, PHB was barely detectable in either of the strains (day 0), while acetate supplementation resulted in a significant increase in the PHB levels by the end of the cultivation period (day 8) (Table 1). Importantly, the changes observed in AT and CS were identical, indicating that enhanced acetate influx does not influence the accumulation of PHB in *Synechocystis* under the conditions tested.

**Table 1:** Quantitative determination of PHB in the *Synechocystis* sp. PCC 6803 acetate transporter expression strain (AT) and the control strain (CS) measured on day 0 and 8, grown in the presence of supplemented 15 mM acetate under continuous light of 20 μmol photons m^−2^ s^−1^ (starting OD_750_ = 0.05)

Strain	Time	PHB (μg L^−1^ OD_750_^−1^)
AT	Day 0	161.7 ± 37.5
	Day 8	3207.0 ± 300.1
CS	Day 0	181.2 ± 27.9
	Day 8	3236.3 ± 201.5

The PHB content was calculated as μg L^−1^ OD_750_^−1^, and mean and standard deviation represent four biological replicates

### The cell size and morphology of the acetate transporter mutant remain unchanged

In order to compare the spectrophotometric properties of the AT mutant and CS, the two strains were analyzed in respect to relative dry cell weight (DCW) and cell size. The DCW measured on day 8 from MC1000 cultivations carried out under low-light conditions in the presence of acetate revealed no significant difference between the strains, and 5 mL of cell culture at OD_750_ = 1 corresponded to about 0.158 g L^−1^ dry cells and 0.154 g L^−1^ dry cells for AT and CS, respectively (Table 2). Microscopic evaluation of the cells supported this finding, and the two strains grown under corresponding conditions appeared identical in size and shape (Table 3). Together the results confirmed that the growth measured based on optical density was not distorted by changes in the cell morphology of the AT mutant, and that OD could be reliably used for normalization between the strains.

**Table 2:** Quantitative determination of dry cell weight of the *Synechocystis* sp. PCC 6803 acetate transporter expression strain (AT) and the control strain (CS) cultivated in the presence of supplemented 15 mM acetate under continuous light of 20 μmol photons m^−2^ s^−1^ (starting OD_750_ = 0.05) for 8 days

Strain	Dry cell weight (g L^−1^)
AT	0.15829 ± 0.01128
CS	0.15350 ± 0.00707

DCW has been calculated as grams per liter culture, and the mean and standard deviation represent four biological replicates

**Table 3:** Comparison of cell sizes between the *Synechocystis* sp. PCC 6803 acetate transporter expression strain (AT) and the control strain (CS) cultivated in the presence of supplemented 15 mM acetate under continuous light of 20 μmol photons m^−2^ s^−1^ (starting OD_750_ = 0.05) for 8 days

Strain	Horizontal cell diameter (arbitrary unit)
AT	0.453 ± 0.0279
CS	0.444 ± 0.0251

The values represent the horizontal diameter of spherical cells measured as arbitrary units, and the data is presented as the mean and standard deviation measured from 115 individual cells of each strain

### ActP expession is linked with reduced photosynthetic activity

**Fig. 4:**
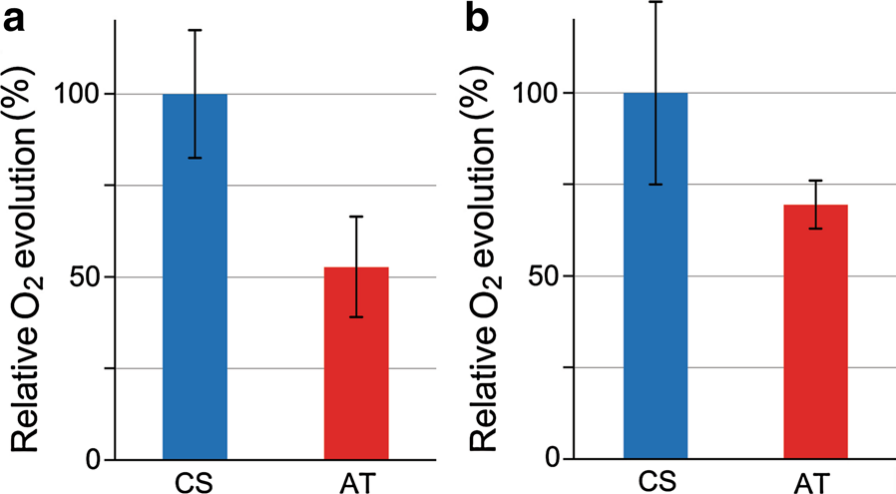
Photosynthetic activity of the cyanobacterial strains. Relative oxygen evolution capacity of *Synechocystis* sp. PCC 6803 acetate transporter expression strain (AT; *red bars*) in comparison to the control strain (CS; *blue bars*) grown for eight days in MC1000 under continuous low light (20 μmol photons m^−2^ s^−1^). **a** Light-saturated oxygen evolution of PSII measured in the presence of artificial electron acceptor DCBQ. **b** Light-saturated whole chain net photosynthesis from water to NaHCO_3_. The oxygen evolution capacity was calculated as µmol O_2_ produced h^−1^ (mL cell culture)^−1^ at OD_750_ = 1. The mean and standard deviation represent three biological replicates

To further understand the metabolic changes induced in the AT mutant under low light in the presence of acetate, oxygen evolution was measured to evaluate the photosynthetic performance of the mutant in comparison to the control strain. Interestingly, even though the growth rate was significantly higher for the AT mutant under the cultivation conditions (Fig. 1e), PSII capacity to produce O_2_ under light-saturated conditions was reduced to 47% in respect to CS (Fig. 4a). In parallel, net oxygen evolution of the AT mutant, measured for the entire chain from water to CO_2_, was only 69% of the control strain (Fig. 4b), demonstrating that augmented intake of acetate had a negative effect on the function of the photosynthetic apparatus under low-light conditions in *Synechocystis*.

### Absorption spectrum of the ActP mutant differs from CS

**Fig. 5:**
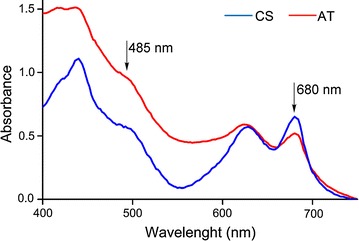
Absorption spectra of the cyanobacterial strains. Absorption spectra (400–750 nm) of the *Synechocystis* sp. PCC 6803 acetate transporter expression strain (AT; *red line*) and the control strain (CS; *blue line*) grown for 8 days in the MC1000 under continuous low light (20 μmol photons m^−2^ s^−1^). The absorption peaks for Chl *a* (680 nm) and carotenoids (485 nm) are indicated by *arrows*. The graphs are representatives of four parallel biological replicates, and have been normalized to 750 nm

To further investigate changes accompanied with the reduced photosynthetic activity in the AT mutant, the absorption spectrum of the AT mutant was evaluated in respect to the control strain at day 8 under low-light growth conditions in the presence of acetate. In accordance to the reduced PSII activity (Fig. 4a), the 680 nm absorption peak for Chl *a* was lower in the AT mutant in comparison to CS (Fig. 5). At the same time, the intracellular carotenoid content (~485 nm) of the AT mutant appeared to be significantly increased (Fig. 5), despite the fact that visual inspection of the cultures did not reveal associated phenotypic color change towards yellow. Based on the absorbance spectrum at 620 nm, the amount of light harvesting phycobilisomes appeared to be rather similar in both strains.

### ActP mutant shows reduced content of reactive oxygen species

**Fig. 6:**
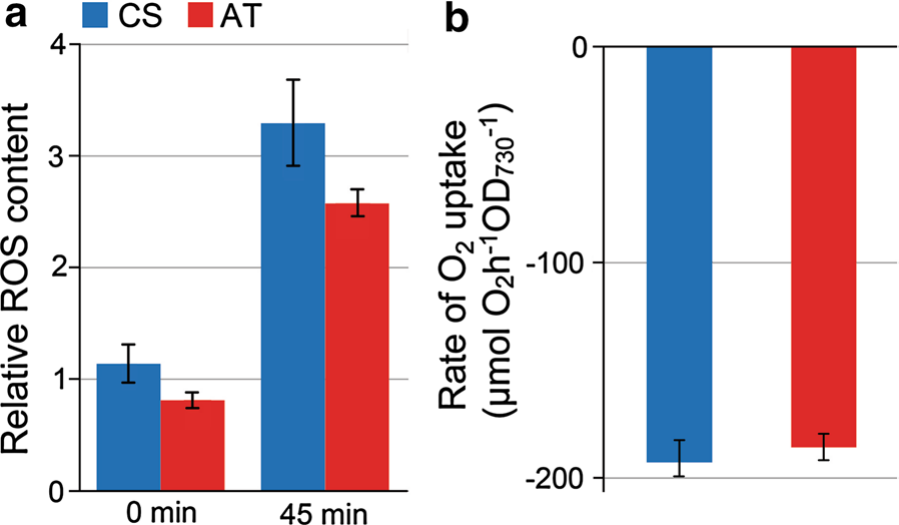
Determination of reactive oxygen species (ROS) in *Synechocystis* sp. PCC 6803 acetate transporter expression strain (AT; red bars) in comparison to the control strain (CS; *blue bars*) grown for 8 days in MC1000 under continuous low light (20 μmol photons m^−2^ s^−1^). **a** Overall cellular ROS content measured immediately (0 timepoint) and after 45 min extension in the standard low light growth conditions using the membrane permeable fluorescence indicator CM-H_2_DCFDA. The mean and standard deviation represent four biological replicates. **b** Production of singlet oxygen in the presence of 5 mM His upon illumination of the cells at 3000 μmol photons m^−2^ s^−1^. The oxygen uptake rate was calculated as µmol O_2_ produced h^−1^ mL^−1^ (cell culture) at OD_750_ = 1.5. The mean and standard deviation represent three biological replicates

To investigate possible reasons for the changes observed in the pigment content and the oxygen evolution capacity of the AT mutant, the analysis of ROS was carried out for the two strains cultivated under low-light growth conditions in the presence of acetate. Intracellular hydrogen peroxide, hydroxyl radicals, peroxyl radicals, and peroxynitrite were measured using the fluorescent oxidative stress indicator CM-H_2_DCFDA. The analysis revealed that AT mutant had 29 and 22% lower ROS content in comparison to CS, as measured under darkness and under standard low-light conditions after 45 min exposure, respectively (Fig. 6a). Singlet oxygen, in turn, was quantitated by measuring His-mediated oxygen uptake of the cells. The assay is based on the interaction between singlet oxygen and the aromatic side chain of His, leading to proportional disposal of dissolved O_2_ from the suspension [18]. Unlike for the other ROS, the analysis showed that there were no detectable differences in the ^1^O_2_ content of the two strains (Fig. 6b).

## Discussion

Due to the absence of the glyoxylate shunt needed for gluconeogenesis, acetate cannot be used as the sole carbon source for heterotrophic growth of the cyanobacterium *Synechocystis* sp. PCC 6803. However, acetate could serve as a supplementary precursor for the biosynthesis of amino acids and lipids, and other metabolites derived from acetyl-CoA. In this study we evaluated the effects of facilitated acetate influx into *Synechocystis* by introduction of a heterologous acetate transporter ActP from *E. coli*, followed by characterization of the resulting phenotypic and biochemical changes in the expression host. The results provide new insight into acetate metabolism in *Synechocystis*, and the possibilities and limitations of using acetate as an additional substrate in the development of future biotechnological applications.

Acetate supplementation into the culture medium did not enhance the growth of WT *Synechocystis* under continuous light and atmospheric CO_2_, in agreement with earlier reports [7]. This implicated that availability of acetate per se does not directly secure acetyl-CoA—derived building-blocks for biomass production, and that the uptake (i.e. incorporation of acetate into the cell metabolism) is determined by some other factors under the specified conditions. However, as a central finding of the present study, expression of the heterologous acetate transporter enzyme ActP resulted in faster growth in the presence of acetate (Fig. 1c–e). This directly demonstrated that (1) the use of acetate by WT *Synechocystis* is at least partially restricted by limited intake into the cell, and that (2) increased intracellular acetate concentration may promote the use of acetyl-CoA and the biosynthesis of the corresponding metabolites. Thus, the native potential of the cells to utilize acetate can be more effectively exploited by over-expressing a transporter which allows facilitated intake. This finding is in contrast with earlier reports suggesting that in the pH range used in the cultivations, extracellular acetate is uncharged and expected to diffuse freely through the cyanobacterial cell membrane [10], while the limiting step would be conversion into acetyl-CoA rather than the transport [16]. The phenotypic growth enhancement of the acetate transporter expression strain was more profound under low-light conditions (Fig. 1e), suggesting that the cell can more effectively take advantage of the increased acetate influx when the photosynthetic activity is limited. This may be linked, at least in part, to the increased availability of precursors for fatty acid and amino acid biosynthesis when the autotrophic metabolism is running slow and photosynthesis-derived acetyl-CoA is scarce. At the same, breakdown of the supplemented acetyl-CoA in the oxidative TCA cycle provides additional energy (reducing equivalents and ATP) for the cell, serving as an advantage under the low-light conditions.

The observed growth phenotype of the AT mutant under low-light and in the presence of acetate appears to be also linked with the glycogen metabolism of the cell; faster growth is accompanied by an overall reduction of intracellular glycogen content as compared to the CS (Fig. 3). Natively, glycogen serves as a carbon and energy reserve which is accumulated in photoactive cyanobacterial cells when carbon is abundant but growth is restricted, for example, by the lack of macronutrients or unfavourable pH [19, 20]. Under normal conditions, glycogen is consumed during dark periods when autotrophic metabolism is not sufficient to sustain necessary cellular functions, and the cell relies on glycolysis for reducing equivalents, ATP and biosynthetic intermediates. As observed for the AT mutant, however, the glycogen content either (1) continues to decrease after the initial lag period (Fig. 3a; normalized to Chl *a*), suggesting that the presence of acetate would induce the mobilization of the existing intracellular carbohydrate reserves for glycolytic growth, or (2) remains constant (Fig. 3b; normalized to cell density), implicating that facilitated acetate influx would inhibit the biosynthesis of glycogen, and thus preventing the restoration of the native levels. Either way, at least some of the energy normally contained in the native glycogen reserves in the CS may be directed to promote photomixotrophic growth of the AT mutant under the low-light conditions when the role of photosynthesis on growth is limited. Thus, even though *Synechocystis* is unable to utilize acetate directly as the sole carbon source, acetate may stimulate biomass formation by altering the carbohydrate metabolism of the cell—which is observed as higher growth rates in the AT mutant. This is in line with the earlier reports that acetate supplementation results in reduced glycogen content even in the WT *Synechocystis* [11], and possibly the upregulation of genes associated with glycolytic metabolism such as phosphoenylpyruvate synthase [21]. In contrast to glycogen, the PHB content of the *Synechocystis* cells appeared not to be affected by enhanced acetate intake. While the intracellular PHB levels increased in presence of acetate as expected [11, 13], no difference was observed between the AT mutant and the control strain (Table 1), implicating that the native acetate intake was sufficient to induce PHB accumulation at full efficiency.

The implication that enhanced acetate influx specifically stimulates the glycolytic growth of *Synechocystis* is further supported by the concomitant reduction of photosynthetic capacity under the tested conditions. This is seen as a drop in PSII oxygen evolution rate down to about 50% (Fig. 4a), significant reduction in the light-saturated whole chain net photosynthesis (Fig. 4b), and decrease in the chlorophyll content (Fig. 5) in the acetate transporter mutant in comparison to the control strain. A corresponding response to acetate supplementation, observed as reduced O_2_ evolution, has also been reported in the WT *Synechocystis* [11]. This may be caused by a direct inhibitory effect of acetate on the photosynthetic apparatus, as small carboxylate anions are known to compete with the bicarbonate/carbonate associated to PSII [22, 23]. In *Chlamydomonas reinhardtii*, for example, acetate has been suggested to inhibit PSII by replacing the bicarbonate associated to the non-heme iron and changing the environment of Q_A_ and Q_B_ which affects photosystem II charge recombination events [21]. In addition to this primary effect, the resulting imbalance in the function of photosystems may lead to enhanced production of intracellular ROS [24]. A characteristic indicator of this is the upregulation of carotenoids, as observed for the acetate transporter mutant (Fig. 5), which acts to protect the cell from the harmful oxidative effects inflicted by ROS on biomolecules such as DNA, proteins and lipids. However, as quantitative analysis of ROS clearly indicated, the total ROS levels in the AT mutant were decreased in comparison to the CS (Fig. 6). This suggests that even if enhanced acetate intake triggers an oxidative stress response in the mutant, the cells are able to adapt to the associated metabolic changes, and do not suffer from ROS in extended exposure to acetate.

**Fig. 7:**
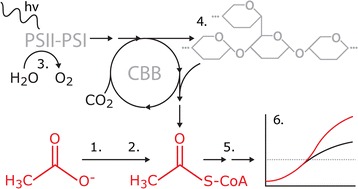
A simplified representation of the metabolic effects associated with enhanced acetate influx in *Synechocystis* sp. PCC 6803 under low-light conditions. Introduction of a heterologous acetate transporter *1* improves the ability of the cell to import supplemented acetate from the medium, which *2* increases the availability of intracellular acetyl-CoA. At the same time, *3* the photosynthetic capacity of the cell is decreased. This is accompanied by *4* the decrease of intracellular glycogen levels, implicating that the available reserves are utilized directly by the cell, instead of storing glucose for future needs. Together this leads to the *5* increased availability of primary precursors for the biosynthesis of, for example, lipids and proteins, as well ATP and reducing equivalents needed to sustain the metabolic reactions. Ultimately, these changes result in *6* enhanced growth of the *Synechocystis* acetate transporter mutant under low-light conditions in the presence of acetate. In the scheme increase/upregulation is depicted in *red* and decrease/downregulation in *grey*

Taken together, the stimulated growth effect, inhibition of photosynthetic electron transfer and reduction of the glycogen content in the acetate transporter mutant are all interlinked (Fig. 7). The low-light conditions and direct inhibitory effect exerted by acetate on the autotrophic metabolism are expected to have an immediate negative effect on the capacity of the cell to accumulate glycogen [19, 25]. At the same time, it appears unlikely that the increased availability of acetyl-CoA alone would account for the observed growth phenotype, and that redirection of part of the energy typically stored in the glycogen reserves could contribute to the improved growth of the expression strain in the presence of acetate. Thus, although biotechnological production systems do not generally aim for biomass formation, enhanced acetate influx could provide a competitive advantage by (1) accelerating the biosynthesis of acetyl-CoA derived metabolites under unoptimal photosynthetic conditions, by (2) serving as an additional energy supply through the TCA cycle, and by (3) modulating the glycogen metabolism to redistribute the available resources for the direct advantage of the cell. Besides providing possible means for improving the production of alcohols [26] and organic acids [7] in cyanobacteria, acetate utilization could be coupled to the production of lipids [27, 28], hydrocarbons [29] or hydrogen [30]—thus making the engineered system of potential biotechnological interest in the design of future applications.

## Methods

### Reagents and enzymes

The restriction enzymes, T4 ligase and DNA polymerase enzymes were obtained from New England BioLabs (Ipswich, MA, USA) or from Thermo-Scientific Fermentas (Finland). Oligonucleotides were from Eurofins MWG Operon (Germany). All chemicals were purchased from Sigma-Aldrich (Finland) if not mentioned otherwise.

### Organisms and growth conditions

*Escherichia coli* strain DH5α was used as the host for the plasmid construction in this study. The cells were grown in Luria-Bertani (LB) medium at 37 °C in a shaker at 150–200 rpm or on solid LB plates containing 1.5% (w/v) agar. When necessary, LB medium was supplemented with appropriate antibiotics at the following concentrations: 50 µg/mL spectinomycin (Spec) and 20 µg/mL streptomycin (Str).

The glucose tolerant substrain of *Synechocystis* sp. PCC 6803 was used for all the cyanobacterial experiments. The cells were grown in liquid BG-11 medium containing 17 mM NaNO_3_ (J. B. Baker) and 20 mM TES-KOH pH 8.0. The AT and CS strains were cultivated in the presence of 25 µg/mL Spec and 12.5 µg/mL Str, and the cultures were supplied with 0.5 mM of isopropyl-β-d-thiogalactopyranoside (IPTG) and 0–30 mM sodium acetate when necessary. All flask cultures were carried out in Algaetron 230 growth chambers (Photon Systems Instruments**)** at 30 °C under continuous light of 20–50 µmol photons m^−2^ s^−1^, and ambient CO_2_ in ~120 rpm orbital shaking. BG-11 plates containing additional 1.5% (w/v) Bactoagar (Difco, USA) and 0.3% (w/v) sodium thiosulfate were used for solid plate cultivations, incubated under corresponding conditions in 1% CO_2_ in SANYO growth chambers.

### ActP construct and expression strains

The acetate transporter gene *yjcG* (*actP*) was amplified by PCR using *E. coli* K-12 substrain MG1655 genomic DNA [14] as a template, and primers *gctc* GGTACCATGAAAAGAGTTCTGACGGCGCTTG and tcagCTGCAGTTAATGCGCGCGGCCTTGCT carrying the *Kpn*I and *Pst*I restriction sites (underlined), respectively. The amplified DNA fragment (1670 bp) was subcloned (*Kpn*I–*Pst*I) into an autonomously replicating expression shuttle vector pDF-lac under the control of an inducible lac-promoter [17] and transformed into *E. coli* DH5α strain for propagation. Wild type *Synechocystis* was transformed with the resulting pDF-lac-yjcG shuttle vector by natural transformation [31], and plated on BG-11 plates supplemented with increasing amount of Spec and Str. The presence of the pDF-lac-yjcG in the antibiotic resistance clones was confirmed by a colony PCR using primer set of forward primer (GCTGCAACTCTCTCAGGGCCAG) and reverse primer (GCGCTTATGGCAGAGCAGGG). The control strain (CS) used in all the experiments was prepared by transforming a corresponding empty plasmid (pDF-trc) [17] into the WT strain.

### Photobioreactor cultivations

Comparison of the *Synechocystis* strains was carried out using a photobioreactor Multi-Cultivator MC1000 (Photon Systems Instrument, Czech Republic), with eight parallel 100 mL culture tubes immersed in a temperature-controlled water bath, each equipped with individual LED lights, aeration, and capacity for automated periodical measurement of optical density at 730 nm. Before each experiment, the MC1000 lights were calibrated with Li-COR (Li-250A Light Meter, Biosciences), and aeration was adjusted (based on bubbling) between the replicates. Precultures prepared in Erlenmeyer flasks were diluted to the optical density of 0.05 or 0.1 at 750 nm, measured in 10 mm single-use cuvettes using a Thermo-Scientific Genesys 10S UV–Vis spectrophotometer, and transferred into MC1000 tubes in the final culture volume 80 mL. To induce expression, 0.5 mM of IPTG was added to the cultures, with 15 mM sodium acetate unless mentioned otherwise. At the end of the cultivations, pH and the wavescans were recorded, and aliquots were streaked on LB-plates to confirm that the cultures were free of contamination. The cultures were typically carried out in at least three biological replicates with two repetitions in each case.

### Evaluating cell size and morphology

For the microscopic evaluation of cell size and morphology, the *Synechocystis* strains were cultured in MC1000 under continuous 20 μmol photons m^−2^ s^−1^ light with supplemented 15 mM acetate for 7 days. The cells were examined under a light microscope (Leitz Orthoplan Large Field Research Microscope) and photographed with a digital microscope camera (Leica DFC420C). The cells were visualized at 100-fold magnification with Leica Application Suite V 4.1, and the horizontal diameter of 115 AT and CS cells were measured form the digital captures (in arbitrary units) using Adobe Photoshop CS4 Ruler Tool.

### Determining the dry cell weight of the *Synechocystis* strains

In order to compare the dry cell weight of AT and CS, the strains were cultivated in MC1000 under 20 μmol photons m^−2^ s^−1^ constant light for 8 days. Aliquots of four biological replicate cultures of each strain were concentrated to the same optical density (OD_750_ = 1), and 5 mL of the samples were filtered through pre-weighted microfiber membranes (VWR). The membranes were washed once with deionized water, oven-dried at 98 °C, and weighted using an analytical scale (Sartorius MC1 Research RC 210P).

### Measuring the use of acetate

The consumption of acetate was measured from 20 mL batch cultures (three biological replicates for each strain) grown in 100 mL Erlenmeyer bottles. The cultures were diluted to the OD_750_ of 0.05, induced with 0.5 mM IPTG, and grown in BG-11 supplemented with antibiotics at ambient air under continuous low light (20 μmol photons m^−2^ s^−1^). Samples were collected on days 0, 2, 4, 6, 8, 10 and 12 followed by sample collection (0.5 mL each) and storage at −80 °C until analysis. Acetate quantitation was carried out from the growth media with Acetate Colorimetric Assay kit (Sigma-Aldrich) according to manufacturer’s instructions.

### Quantification of glycogen

For quantitative glycogen analysis, 1 mL cell samples (three biological replicates for each strain) were collected from MC1000 cultivations grown under 20 μmol photons m^−2^ s^−1^ at the time points 0, day 4, day 8 and day 10. The quantitation was carried out with a commercial Total Starch Assay Kit (Megazyme) using a modified method [32]. The collected samples were pelleted and resuspended in 100 µL of 50 mM NaOAc + 5 mM CaCl_2_ pH 5.0. The cells were lysed by addition of 900 µL ethanol (96%) and acid washed glass beads (Sigma), followed by eight cycles of vortexing (1 min) and incubating on ice (2 min). The lysates were incubated at 90 °C for 10 min, on ice for 30 min, and centrifuged at 16000*g* for 30 min. The supernatant was used for chlorophyll *a* quantitation, while the pellets were incubated at 45 °C to remove the ethanol. Subsequently, the pellets were resuspended into 100 µL of 50 mM NaOAc + 5 mM CaCl_2_ pH 5.0-buffer with 0.75 U of α-amylase and 5 U of amyloglucosidase to break down the glycogen into glucose. The samples were mixed well and incubated at 60 °C for 2 h, followed by the addition of 150 µL of supplied GOPOD-reagent into the samples. The amount of released glucose in the cell was determined by colourimetric method by reading the absorbance at 510 nm with a PlateReader (Tecan infinite 200 PRO).

### Quantification of PHB

For quantitative PHB analysis, aliquots of AT and CS cells were collected at the beginning of the main culture and on day 8 of MC1000 cultivation grown under 20 μmol photons m^−2^ s^−1^ with supplemented 15 mM acetate. The PHB quantitation was carried out for four biological replicates for each strain, using a commercial D-3-Hydroxybutyric Acid Assay Kit (Megazyme) according to manufacturer’s protocol. The samples were first adjusted to correspond to OD_750_ = 0.1 in 50 mL total volume. After centrifugation, the pellets were suspended to 400 µL of 0.5 M NaOH and incubated for 1 h at 85 °C in shaking. The samples were then cooled down and neutralized with 100 µl of 1 M HCl, and the generated D-3-hydrozybutyric acid (corresponding to the amount of PHB in the cells) was analysed colourimetrically from the supernatant by recording the absorbance at 492 nm with a PlateReader (Tecan infinite 200 PRO).

### Determination of in vivo light-saturated photosynthetic activity

Samples for the oxygen evolution experiments (three biological replicates for each strain) were collected from MC1000 cultures cultivated under continuous light 20 μmol photons m^−2^ s^−1^ for 8 days, followed by concentration of the cells to OD_750_ 1.5. A Clark-type oxygen electrode (Hansatech Ltd) was used to measure the oxygen evolution capacity of photosystem II (PSII) from 1 mL samples under saturating light (2000 μmol photons m^−2^ s^−1^) at 30 °C in the presence of an electron acceptor 0.5 mM 2,6-dichloro-p-benzoquinone (DCBQ) and 0.5 mM ferricyanide to maintain DCBQ in oxidized form. The light-saturated whole chain net photosynthesis was measured similarly in the presence of 10 mM NaHCO_3_ as carbon source. The values were calculated as µmol O_2_ produced h^−1^ mL^−1^ (cell culture) at OD_750_ = 1.

### Absorption spectra

Absorption spectra (400–800 nm) of all cultures were recorded at the end of each MC1000 cultivation batch using a microplate reader (TECAN Infinite M200PRO). The data was normalized to 750 nm.

### Measuring the cellular ROS content

For determining the overall ROS content of the cells, the AT mutant and CS (four biological replicates for each strain) were cultured in MC1000 under 20 μmol photons m^−2^ s^−1^ with supplemented 15 mM acetate for 8 days. The analysis was carried out using a commercial membrane-permeable fluorescence indicator 5-(and-6)-chloromethyl-2′,7′-dichlorodihydrofluorescein diacetate, CM-H_2_DCFDA (Invitrogen), which detects hydrogen peroxide, hydroxyl radicals, peroxyl radicals, and peroxynitrite, with a protocol modified from Hakkila et al. 2014 [33]. The cells were washed once and resuspended in 1 mL BG-11 supplemented with 15 mM acetate, to OD_750_ 1. Samples were incubated with and without 25 µM CM-H_2_DCFDA for 90 min under darkness at 30 °C with shaking. After washing cells twice the samples were resuspended to the final volume of 0.5 mL. Fluorescense (485 nm excitation and emission 535 nm) and autofluorescence (680 nm; chlorophyll) were measured using a PlateReader (Tecan infinite 200 PRO) on a 96-well microtiter plate (Perkin Elmer Isoplate™ −96 F, Black frame and Clear well). Subsequently, samples were incubated further for 45 min under 20 μmol m^−2^ s^−1^ of light with shaking, and fluorescence was re-measured. Autofluorescence was used for normalization, and changes in CM-H_2_DCFDA fluorescence were used to calculate the overall ROS content of the cells.

### Singlet oxygen detection

The amount of singlet oxygen in the cells was determined by measuring His-mediated oxygen uptake, which is based on the removal of oxygen from the suspension due to oxidation of histidine by ^1^O_2_ [18]. Samples of AT and CS (three biological replicates for each strain) were taken from MC1000 cultures grown under 20 μmol photons m^−2^ s^−1^ with supplemented 15 mM acetate on day 8. The cells were washed once and concentrated to OD_750_ 1.5. The rate of singlet oxygen-induced oxygen uptake was measured using Hansatech DW2 O_2_ electrode. Measurements were performed in the presence and absence of 5 mM His under 3000 μmol photons m^−2^ s^−1^ constant light.

## Additional files

**Additional file 1.** Verification of the acetate transporter expression strain. Colony PCR verification of the engineered *Synechocystis* sp. PCC 6803 acetate transporter ActP expression strain (AT) in comparison to the control strain (CS) as visualized by agarose gel electrophoresis. The expected sizes of the PCR fragments were 2.7 Kb for the AT construct harboring *actP* (left lane and middle lane) and 1.1 Kb the empty CS construct (right lane).

**Additional file 2.** Biological replicates of the representative growth curves shown in Figure 1, together with an additional pane (i) showing AT (red line) and CS (blue line) cultivated under continuous light of 50 μmol photons m^−2^ s^−1^ for 10 d (starting at OD_750_ = 0.05) as in “d” without acetate (solid line) and in the presence of 15 mM acetate (dashed line). The growth curves presented in each of the panes a-i are parallels from the same MC1000 cultivation.

## Abbreviations

ACK: acetate kinase
ACS: acetyl-CoA synthetase
AT: acetate transporter expression strain
Chl *a*: chlorophyll *a*
CS: control strain
d: day
DCBQ: 2,6-dichloro-p-benzoquinone
DCW: dry cell weight
IPTG: isopropyl-β-d-1-thiogalactopyranoside
LB: Luria–Bertani
PHB: polyhydroxybutyrate
OD: optical density
PTA: phosphotransacetylase
ROS: reactive oxygen species
Spec: spectinomycin
Str: streptomycin
*Synechocystis*: *Synechocystis* sp. PCC 6803
TCA: oxidative tricarboxylic cycle
WT: wild type
