# Supplementary material for: The effect of enhanced acetate influx on Synechocystis sp. PCC 6803 metabolism
Source: Microb Cell Fact. 2017 Feb 2;16:21. doi: 10.1186/s12934-017-0640-x (PMC5290672; doi:10.1186/s12934-017-0640-x)
Supplement: Supplementary file 1 — Additional file 1. Verification of the acetate transporter expression strain. Colony PCR verification of the engineered Synechocystis sp. PCC 6803 acetate transporter ActP expression strain (AT) in comparison to the control strain (CS) as visualized by agarose gel electrophoresis. The expected sizes of the PCR fragments were 2.7 Kb for the AT construct harboring actP (left lane and middle lane) and 1.1 Kb the empty CS construct (right lane). [file 12934_2017_640_MOESM1_ESM.pdf]

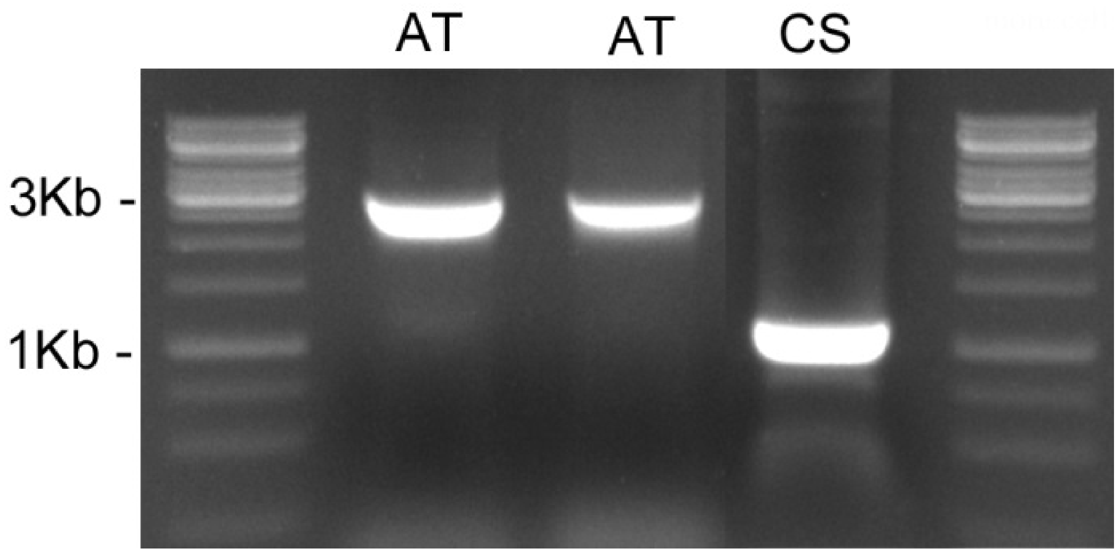

Additional file 1: Verification of the acetate transporter expression strain. Colony PCR verification of the engineered *Synechocystis* sp. PCC 6803 acetate transporter ActP expression strain (AT) in comparison to the control strain (CS) as visualized by agarose gel electrophoresis. The expected sizes of the PCR fragments were 2.7 Kb for the AT construct harboring *actP* (left lane and middle lane) and 1.1 Kb the empty CS construct (right lane).
