# Supplementary material for: The effect of enhanced acetate influx on Synechocystis sp. PCC 6803 metabolism
Source: Microb Cell Fact. 2017 Feb 2;16:21. doi: 10.1186/s12934-017-0640-x (PMC5290672; doi:10.1186/s12934-017-0640-x)
Supplement: Supplementary file 2 — Additional file 2. Biological replicates of the representative growth curves shown in Figure 1, together with an additional pane (i) showing AT (red line) and CS (blue line) cultivated under continuous light of 50 μmol photons m−2 s−1 for 10 d (starting at OD750 = 0.05) as in “d” without acetate (solid line) and in the presence of 15 mM acetate (dashed line). The growth curves presented in each of the panes a-i are parallels from the same MC1000 cultivation. [file 12934_2017_640_MOESM2_ESM.pdf]

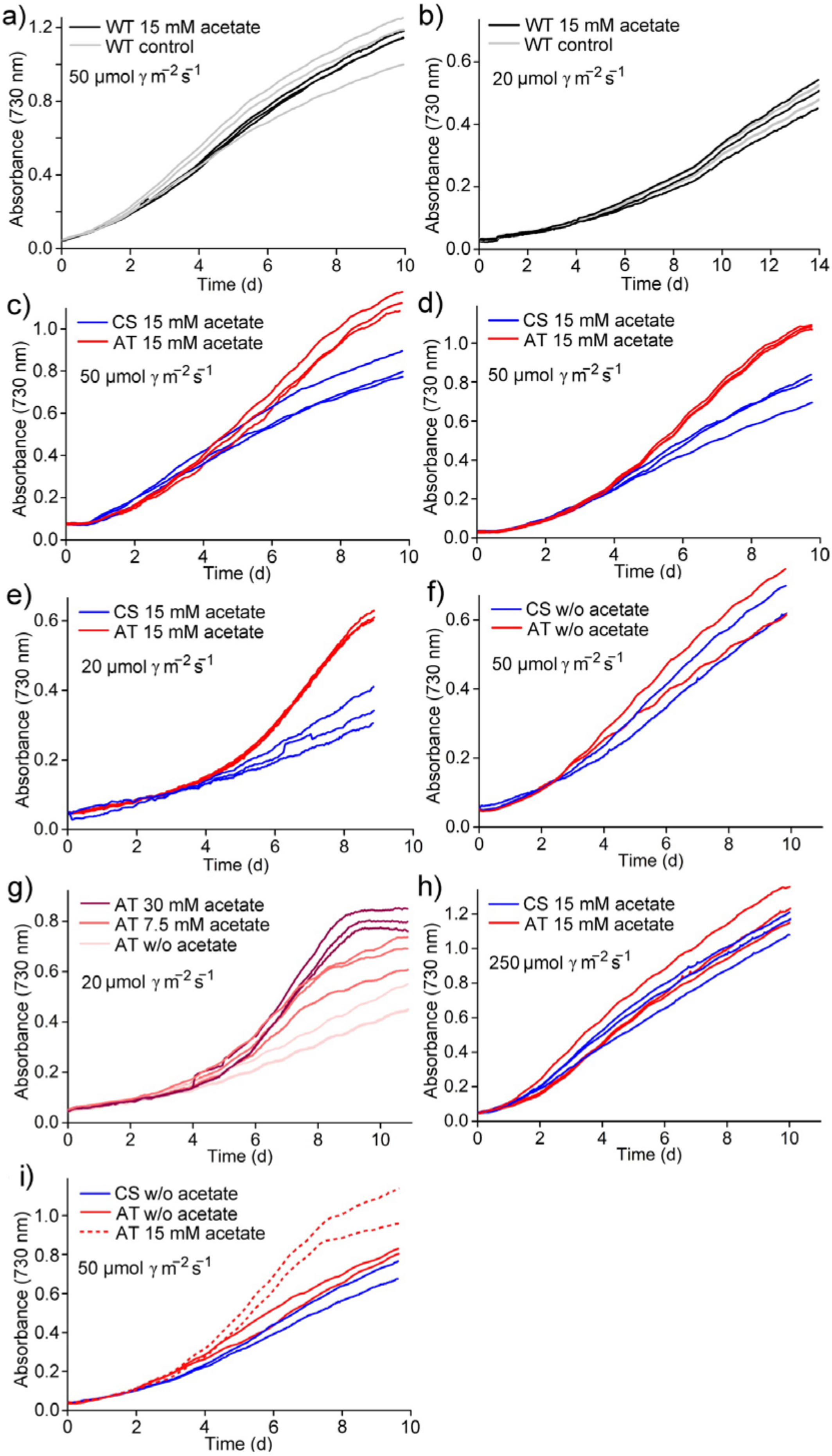

a)
WT 15 mM acetate
WT control
50 µmol γ m-2 s-1
Absorbance (730 nm)
Time (d)
b)
WT 15 mM acetate
WT control
20 µmol γ m-2 s-1
Absorbance (730 nm)
Time (d)
c)
CS 15 mM acetate
AT 15 mM acetate
50 µmol γ m-2 s-1
Absorbance (730 nm)
Time (d)
d)
CS 15 mM acetate
AT 15 mM acetate
50 µmol γ m-2 s-1
Absorbance (730 nm)
Time (d)
e)
CS 15 mM acetate
AT 15 mM acetate
20 µmol γ m-2 s-1
Absorbance (730 nm)
Time (d)
f)
CS w/o acetate
AT w/o acetate
50 µmol γ m-2 s-1
Absorbance (730 nm)
Time (d)
g)
AT 30 mM acetate
AT 7.5 mM acetate
AT w/o acetate
20 µmol γ m-2 s-1
Absorbance (730 nm)
Time (d)
h)
CS 15 mM acetate
AT 15 mM acetate
250 µmol γ m-2 s-1
Absorbance (730 nm)
Time (d)
i)
CS w/o acetate
AT w/o acetate
AT 15 mM acetate
50 µmol γ m-2 s-1
Absorbance (730 nm)
Time (d)

Additional file 2: Biological replicates of the representative *Synechocystis* sp. PCC 6803 growth curves shown in Figure 1. Wild type strain (WT) grown in the presence of 15 mM supplemented acetate (black line) and in the absence of acetate (grey line) **a)** under continuous light of 50 µmol photons m^{-2} s^{-1} for 10 d and **b)** under continuous light of 20 µmol photons m^{-2} s^{-1} for 14 d. Acetate transporter expression strain (AT; red line) and control strain (CS; blue line) grown with 15 mM supplemented acetate starting from **c)** OD_{750} = 0.1 and **d)** OD_{750} = 0.05 under continuous light of 50 µmol photons m^{-2} s^{-1} for 10 d. **e)** AT and CS grown under continuous light of 20 µmol photons m^{-2} s^{-1} for 9 d with 15 mM supplemented acetate. **f)** AT and CS grown in the absence of acetate or IPTG induction under continuous light of 50 µmol photons m^{-2} s^{-1} for 11 d. **g)** AT cultivated with supplemented with 30 mM acetate (dark red line), 7,5 mM acetate (red line) and no acetate (light red line) under continuous light of 20 µmol photons m^{-2} $s^{-1}$for 11 d. **h)** AT and CS grown for 11 d under continuous light of 250 µmol photons m^{-2} s^{-1} with 15 mM supplemented acetate. An additional pane **i)** not included in Figure 1, showing AT (red line) and CS (blue line) cultivated under continuous light of 50 µmol photons m^{-2} s^{-1} for 10 d (starting at OD_{750} = 0.05) as in “d” without acetate (solid line) and in the presence of 15 mM acetate (dashed line). All cultures were started from OD_{750} = 0.05, and AT and CS were grown in the presence of IPTG, unless stated otherwise. AT and CS were always cultivated with 25 µg/ml Spec and 12,5 µg/ml Str to maintain the selection pressure for the expression plasmid (c-i), which resulted in slower overall growth in comparison to WT cultured without antibiotics (a-b). The growth curves presented in each of the panes a-i are parallels from the same MC1000 cultivation.
